# Online Sexual Harassment, Disordered Eating Attitudes, and Body Shame in Young Women

**DOI:** 10.1177/10778012251338476

**Published:** 2025-04-30

**Authors:** Rhea Milson, Ebony Ashton-Rowling, Victoria Cole, Bethan Graham, Aeryn Nicoll

**Affiliations:** 1 School of Education, Language, and Psychology, 41872 York St John University, York, UK

**Keywords:** online sexual harassment, sexual objectification, body shame, eating attitudes, eating disorders

## Abstract

Sexual harassment is associated with disordered eating in young women, directly and indirectly, via body shame. However, today, young women are not only experiencing sexual harassment in-person, but also online. We recruited a non-clinical sample of young women (*N* = 146) to examine the direct and indirect relationships between online sexual harassment, disordered eating attitudes, and body shame. Findings revealed that online sexual harassment positively predicted disordered eating attitudes directly and indirectly, via body shame. These findings offer initial support for examining the utility of online sexual harassment and body shame to further understand disordered eating in young women.

## Introduction

In the United Kingdom, it is estimated that women aged 16–34 years are most likely to experience some form of sexual harassment, with 39% of this demographic reporting a sexual harassment experience in the previous 12-month period (Office for National Statistics, 2023). Research suggests that experiences of sexual harassment are associated with various psychological and well-being outcomes in young women, including anxiety and depression (Agarwal, 2022; Farmer et al., 2025), and most prominently, disordered eating (Hayes et al., 2021). Research also suggests that the association between sexual harassment and disordered eating in women is influenced by body shame (Hayes et al., 2021). However, women are not only experiencing sexual harassment in-person, but also online, via the internet and mobile devices (Gewirtz-Meydan et al., 2024; Iroegbu et al., 2024). In the present study, we are interested in examining online sexual harassment, and whether sexual harassment in this online context predicts disordered eating in young women, and whether this relationship is influenced by body shame.

### Sexual Harassment

Sexual harassment is commonly defined as an experience of any unwanted sexual behavior, including sexual comments, jokes, and gestures (Hayes et al., 2021). Previous research varies in its depiction of sexual harassment, with some studies defining sexual harassment within the boundaries of a particular setting and some research considering sexual harassment more generally across social settings (Berdahl & Raver, 2011; Huerta et al., 2006). Historically, research has focused on sexual harassment in workplace settings (Fitzgerald et al., 1988; Siegel, 2004). However, more recently, research has examined sexual harassment across multiple settings, particularly following the #MeToo movement, which highlighted the prevalence of sexual harassment and assault today (Drewett et al., 2021; Stubbs-Richardson et al., 2024).

Sexual harassment is associated with various psychological and well-being outcomes, such as anxiety, depression, and low self-esteem (Agarwal, 2022; Farmer et al., 2025). However, most prominently, sexual harassment is associated with body image and disordered eating, due to the appearance-focused nature of sexually harassing comments and gestures, which can lead to a preoccupation and dissatisfaction with appearance, in line with objectification theory.

### Sexual Objectification

Sexual objectification is defined as the process of reducing women to their bodies, body parts, or bodily functions, often in a sexualized way, with the misconception that women can be wholly represented by their body parts (Chau et al., 2024). Objectification theory (Fredrickson & Roberts, 1997) posits that sexual objectification can manifest in several ways, from the “objectifying gaze,” whereby a woman's body is sexually observed and evaluated, to more extreme forms of interpersonal sexual objectification, such as explicit sexual advances and sexual harassment.

Objectification theory considers self-objectification as an underpinning mechanism to explain the effect of sexual objectification on women's mental health. Self-objectification is defined as the act of internalizing a cultural ideal standard and evaluating oneself against that standard (Saunders et al., 2024). In Western cultures, the ideal body standards are ever-changing, ranging from the thin-ideal (i.e., a slender body type with small waist and flat stomach; McComb & Mills, 2021; Volonté, 2019) to the fit-ideal (i.e., a toned and athletic body type; McComb & Mills, 2021). More recently, the slim-thick ideal has become popularized (i.e., a curvier body type, characterized by a small waist and flat stomach and larger breasts and hips; McComb & Mills, 2021). Women experiencing self-objectification in relation to these differing (and often contradictory) standards, are likely to internalize these ideals and engage in body surveillance behaviors (i.e., the routine monitoring of one's body and physical appearance and comparing one's body to the ideal standards; Cannavò et al., 2024). According to objectification theory (Fredrickson & Roberts, 1997), this self-objectification and surveillance process often results in women experiencing body shame.

### Body Shame

Shame is a powerful emotion, characterized by a negative evaluation about oneself (Tracy & Robins, 2004). More specifically, body shame is defined as shame felt toward one's body, due to internalized beliefs that one does not meet cultural body ideals (Nechita et al., 2021). Body shame is believed to contribute toward a range of mental health issues for women, particularly issues around eating, as body image concerns are a prominent risk factor for the development of eating disorders (Matos et al., 2023; Nechita et al., 2021; Warnick et al., 2022). This is because the pursuit of body ideals can result in women engaging in unhealthy behaviors, such as dieting and compensatory behaviors (e.g., vomiting, misuse of laxatives), as a means of controlling their weight and aligning closer to these body ideals. Such behaviors can lead to the onset of eating disorders (Stewart et al., 2022; Yuan et al., 2024).

### Disordered Eating

Estimates suggest over 1.25 million people in the United Kingdom have an eating disorder (BEAT, 2020). Eating disorders refer to a group of syndromes characterized by disturbed attitudes and behaviors relating to eating and body image, which lead to adverse effects on mental and physical health, and in some cases, increased mortality (Galmiche et al., 2019). It is considered that eating disorders predominantly affect women in Western cultures (National Institute for Health and Care Excellence, 2020), with incidence rates in young women increasing since the COVID-19 pandemic (Hyam et al., 2023; Trafford et al., 2023). Estimated lifetime prevalence rates of clinically diagnosed eating disorders in women are conservative (i.e., 4% Anorexia Nervosa, 3% Bulimia Nervosa; Van Eeden et al., 2021). However, post-COVID, over 72% of 20- to 25-year-old women were “screened positive” for possible eating problems (NHS Digital, 2023). Moreover, women who do not meet diagnostic criteria but still experience disordered eating symptoms (i.e., sub-clinical populations) may experience the accompanying pathology of individuals with clinically diagnosed eating disorders (Johnson et al., 2021). Therefore, early detection of disordered eating attitudes and behaviors (i.e., in non-clinical samples), and examination of relevant predictors (i.e., sexual harassment and body shame), may help to prevent young women from developing clinically diagnosed eating disorders (Koreshe et al., 2023).

### Sexual Harassment, Body Shame, and Disordered Eating

Hayes et al. (2021) reviewed the existing literature examining the relationship between sexual harassment and eating disorder psychopathology, with most studies recruiting young female samples, and using the Eating Attitudes Test-26 (Garner et al., 1982) to examine disordered eating. Hayes et al. (2021) found that sexual harassment was significantly associated with eating disorder psychopathology (see the following examples, Buchanan et al., 2013; Harned, 2000; Petersen & Hyde, 2013; Romito et al., 2019). This review also examined potential mediating factors in the relationship between sexual harassment and eating disorder psychopathology and found body shame (as measured by the Objectified Body Consciousness Scale-Body Shame Subscale; McKinley & Hyde, 1996), to be a significant mediator (see Holmes & Johnson, 2017; Petersen & Hyde, 2013). These findings offer support for objectification theory, and how body shame may help to explain the relationship between sexual harassment and eating disorder psychopathology in young women.

### Online Sexual Harassment

Theory and evidence suggest that women are at risk of experiencing in-person sexual harassment and the consequential body image and disordered eating outcomes. However, growth in technology and social media has given rise to a new environmental context for sexual harassment (Chau et al., 2024; Gewirtz-Meydan et al., 2024; Iroegbu et al., 2024). Online sexual harassment (also known as cyber sexual harassment) is defined as an experience involving unwanted sexual advances, sexual threats, or sexual attention, experienced through electronic means, such as a mobile phone or via the internet. This form of sexual harassment can include experiences such as unwanted sexual comments or requests to engage in sexual behavior, and unwanted sending or receiving of private or explicit digital content (Buchanan & Mahoney, 2022; Iroegbu et al., 2024). Like in-person sexual harassment, research suggests women are also more likely to experience online sexual harassment than men (Chau et al., 2024, Iroegbu et al., 2024), particularly young women under the age of 30 years (Cuenca-Piqueras et al., 2020; Salerno-Ferraro et al., 2022).

Although online sexual harassment can be defined as an extension of in-person sexual harassment, or as a similar construct but within a different context (Burnay et al., 2019; Van Royen et al., 2015), it is important to note the distinctions between the two forms of sexual harassment. In contrast to in-person interactions, the online environment can promote greater anonymity, invisibility, and disinhibition effects, whereby men may express hostility in their views toward women and behave in a deviant manner online, to a greater extent than they would offline (Angela et al., 2023; Buchanan & Mahoney, 2022; Rosemary et al., 2024). Moreover, although some sexual harassment behaviors can occur both online and offline (such as unwanted sexual comments), some sexual harassment behaviors may relate exclusively to online contexts, such as the non-consensual sharing of sexual images or videos, and “revenge pornography” (whereby, an individual shares explicit content of their partner via online messaging or internet sites, typically following the breakup of a relationship; Bates, 2017; Branch et al., 2017; Drouin et al., 2015). The speed at which content can be shared and messages can be sent, and the potential permeance of these behaviors, highlights the nuance and specificity of sexual harassment in an online context, and the importance of investigating online sexual harassment and its psychological outcomes. Furthermore, the surge in online sexual harassment experiences reported by women since the COVID-19 pandemic highlights the relevance and importance of investigating sexual harassment specifically within an online context (Ahuja & Padhy, 2021; Huiskes et al., 2022).

### Online Sexual Harassment, Body Shame, and Disordered Eating

In line with objectification theory (Fredrickson & Roberts, 1997), women who repeatedly experience sexual harassment online may internalize this sexual objectification (i.e., self-objectification), start to habitually evaluate their body and appearance (i.e., body surveillance), and start to feel shame toward their body if they believe they do not meet the expected standards (i.e., body shame). This body shame may then result in disordered eating attitudes and behavior, as a means of trying to achieve these body ideals. Oliver et al. (2024) support this, reporting that women who experience a greater frequency of online sexual harassment, also report greater levels of disordered eating (as measured by the Eating Attitudes Test-26). However, body shame was not investigated in this previous study, therefore, the specific role of body shame within this relationship is unclear. Dollimore et al. (2025) expanded on this, reporting that online sexual harassment was directly associated with body shame. However, the resulting associations with disordered eating were not examined. Therefore, although online sexual harassment has been examined in relation to disordered eating, and in relation to body shame, there is limited research examining all variables within one study, i.e., examining the specific role of body shame within the relationship between online sexual harassment and disordered eating.

### The Present Study

To expand on the existing literature, we aimed to examine the direct and indirect relationships between online sexual harassment, disordered eating attitudes, and body shame in young women. Based on theory and research, we expected that both online sexual harassment and body shame would be positive predictors of disordered eating attitudes and that there would be an indirect association between online sexual harassment and disordered eating attitudes, via body shame.

## Method

### Participants

Participants were 146 women from the United Kingdom, aged 18–30 years (*M* = 19.14, *SD* = 1.69). Recruitment over a 2-month period was by opportunity sampling, via social media platforms (Facebook, Instagram, and X—formerly known as Twitter) and York St John University's research participation scheme, whereby students could take part in the study for course credit. Participants who did not identify as female were excluded from the study. Participants with a formal diagnosis of an eating disorder or body image disorder were also excluded from the study, via self-report screening, as we were interested in recruiting non-clinical samples, to examine factors that may help to prevent young women developing clinical disorders, rather than treating current disorders. A priori power analysis, using G*Power, was conducted to determine the sample size required for the correlational analysis. Using a previous meta-correlation of .29 (Mason et al., 2021), a power of .80 (Abraham & Russell, 2008; Bakker et al., 2016) and an alpha of .05 (Miller & Ulrich, 2019), the power analysis estimated that a minimum sample of 88 participants would be required.

### Measures

#### Online Sexual Harassment

We used the Online Sexual Harassment Scale (Buchanan & Mahoney, 2022) to measure online sexual harassment victimization. This measure consisted of 12 items, examining unwanted experiences while using the internet or a mobile device within the past 12 months (e.g., “Have you received an unwanted explicit sexual message or text?”) A 5-point, bipolar, Likert scale was used for each item, ranging from *never* (0) to *all the time* (4). Buchanan and Mahoney (2022) reported an overall scale reliability of ω .95. Cronbach's alpha was calculated to assess scale internal reliability for the present study (α = .95).

#### Disordered Eating Attitudes

We used the Eating Attitudes Test-26 (Garner et al., 1982) to measure disordered eating attitudes. This measure consisted of 26 items, examining self-reported symptoms and concerns that are characteristic of eating disorders (e.g., “I am terrified about being overweight”). A 6-point, bipolar, Likert scale was used for each item. For Items 1–25, the scale ranged from *always* (3) to *never* (0) and for Item 26, the scale ranged from *never* (3) to *always* (0). Garner et al. (1982) reported that this measure is highly correlated with the original Eating Attitudes Test-40 measure (*r* = .98) and has high internal consistency as an overall measure (α = .90). Cronbach's alpha was calculated to assess scale internal reliability for the present study (α = .88).

#### Body Shame

We used the Objectified Body Consciousness Scale-Body Shame Subscale (McKinley & Hyde, 1996) to measure body shame. This measure consisted of eight items, examining body shame and the internalization of cultural beauty standards (e.g., “I would be ashamed for people to know what I really weigh”). A 7-point, bipolar, Likert scale was used for each item, ranging from *strongly disagree* (1) to *strongly agree* (7). McKinley and Hyde (1996) reported that this subscale has high internal consistency (α = .84) for undergraduate women. Cronbach's alpha was calculated to assess scale internal reliability for the present study (α = .86).

### Procedure

Ethical clearance for all procedures was approved by the relevant ethics committee. Informed consent was obtained for all participants and questionnaires were distributed on Qualtrics, via social media platforms (Facebook, Instagram, and X—formerly known as Twitter) and the York St John University research participation scheme.

### Analytic Strategy

We first conducted preliminary analysis, including screening for outliers. Next, we calculated descriptive statistics and bivariate correlations.

Regression and analysis of indirect association were then used to examine the direct and indirect relationships between online sexual harassment, disordered eating attitudes, and body shame.

## Results

### Descriptive Statistics and Correlations

The data were analyzed using IBM SPSS Statistics 29. First, we screened the data for univariate and multivariate outliers. One univariate outlier was detected (i.e., standardized scores that were greater than *z* = 3.29) and this participant was removed from further analyses. Descriptive statistics and bivariate correlations are presented in Table 1.

**Table 1.** Bivariate Correlations, Means, and Standard Deviations.

Variable	1	2	3
Online sexual harassment	—		
Body shame	.231*	—	
Disordered eating attitudes	.321**	.612**	—
*M*	28.14	34.62	15.37
*SD*	11.76	10.17	11.81

*Note. N* = 146.

* *p* < .01. ** *p* < .001.

### Regression and Analysis of Indirect Association

Next, we conducted regression analyses to examine how online sexual harassment predicted disordered eating attitudes (Model 1), how body shame predicted disordered eating attitudes (Model 2), and how the combination of online sexual harassment and body shame predicted disordered eating attitudes (Model 3). To account for the sample size, we have reported adjusted *R*^2^.

Model 1 accounted for 9.7% of the variance in disordered eating attitudes, *R*^2^ = .097, *F*(1, 144) = 16.53, *p* < .001. In Model 1, online sexual harassment made a significant contribution to the regression model (β = .321, *p* < .001).

Model 2 accounted for 37.0% of the variance in disordered eating attitudes, *R*^2^ = .370, *F*(1, 144) = 86.09, *p* < .001. In Model 2, body shame made a significant contribution to the regression model (β = .612, *p* < .001).

For Model 3, we examined the combination of online sexual harassment and body shame in one model. In Step 1, we added online sexual harassment and in Step 2, we added body shame. Model 3 accounted for 40.0% of the variance in disordered eating attitudes, *R*^2^ = .400, *F*(2, 143) = 49.32, *p* < .001. Prior to adding body shame into the model, online sexual harassment made a significant contribution to the regression model (β = .321, *p* < .001). When body shame was added into the model, the contribution of online sexual harassment to the model reduced (β = .190, *p* = .005), suggesting an indirect association between online sexual harassment and disordered eating attitudes, via body shame (i.e., online sexual harassment → body shame → disordered eating attitudes), and indicating provisional support for a partial mediating effect (Baron & Kenny, 1986). However, potential mediation is tentative at this stage, and longitudinal data would be required to test true mediation (Jose, 2016).

**Figure 1.**
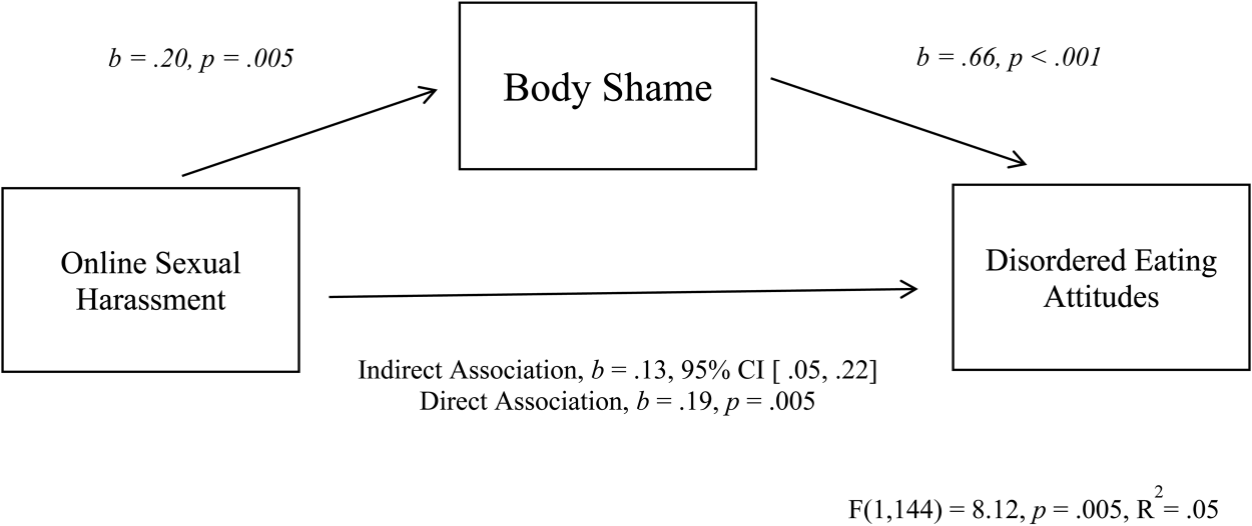
Indirect association between online sexual harassment and disordered eating attitudes, via body shame.

To further test the indirect association between online sexual harassment and disordered eating attitudes, via body shame, we examined the size and significance of this indirect association using PROCESS Model 4 (Hayes, 2013), running a model with 5,000 bootstraps. If the 95% confidence interval (CI) does not contain zero, the test can be considered significant at the *p* < .05 level (Preacher & Hayes, 2008). In line with expectations, results confirmed that the indirect association was significant (indirect association = 0.13, 95% CI [.05, .22]; see Figure 1).

Overall, these findings suggest that online sexual harassment is directly associated with disordered eating attitudes in young women, and indirectly associated, via body shame.

## Discussion

We examined the direct and indirect relationships between online sexual harassment, disordered eating attitudes, and body shame in young women. We found that online sexual harassment positively predicted disordered eating attitudes, and body shame positively predicted disordered eating attitudes. We also found that the combination of online sexual harassment and body shame predicted disordered eating attitudes, indicating an indirect association between online sexual harassment and disordered eating attitudes, via body shame.

We expected that young women who reported experiences of online sexual harassment would also report greater disordered eating attitudes. We found support for this, as online sexual harassment positively predicted disordered eating attitudes. This aligns with previous cross-sectional work in the context of in-person sexual harassment (Romito et al., 2019; Zheng & Lyu, 2024). Notably, Hayes et al. (2021) systematically reviewed the existing literature and found that in-person sexual harassment was significantly associated with eating disorder psychopathology in young women. Previous work has also examined the relationship between online sexual harassment and disordered eating, suggesting consistent findings for both forms of sexual harassment (Oliver et al., 2024). Therefore, the findings from the present study are consistent with the previous work examining in-person sexual harassment and online sexual harassment, and its association with disordered eating in young women.

We also expected that young women who reported greater levels of body shame would also report greater disordered eating attitudes. We found support for this, as body shame positively predicted disordered eating attitudes. Previous research supports that body shame is a key predictor of disordered eating symptoms, within the framework of objectification theory (O'Loghlen et al., 2022). Notably, a meta-analysis of 195 studies by Nechita et al. (2021) reported that body shame was strongly associated with eating disorder symptoms. The findings from the present study are therefore consistent with previous research that has reported an association between body shame and disordered eating in young women.

Based on well-researched general principles regarding the indirect role of body shame in the relationship between in-person sexual harassment and disordered eating (Fredrickson & Roberts, 1997; Kozee et al., 2007), we further expected that there would be an indirect association between online sexual harassment and disordered eating attitudes, via body shame. We found support for this, as when body shame was combined with online sexual harassment, the contribution of online sexual harassment reduced, suggesting an indirect association between online sexual harassment and disordered eating attitudes, via body shame. Previous research has examined the indirect role of body shame in the relationship between in-person sexual harassment and disordered eating. Holmes and Johnson (2017) reported that the relationship between sexual victimization (i.e., unwanted sexual experiences) and disordered eating in women, was mediated by body shame. Furthermore, in a systematic review by Hayes et al. (2021), body shame was reported as a significant mediator in the relationship between in-person sexual harassment and disordered eating, in women. The findings from the present study are not only consistent with previous work examining in-person sexual harassment, but also further the research area, by suggesting that body shame plays an indirect role in the relationship between disordered eating and sexual harassment in an online context. Previous research offers partial support for this online context, as Dollimore et al. (2025) reported that online sexual harassment was directly associated with body shame. However, the resulting associations with disordered eating were not examined. Therefore, although online sexual harassment has been examined in relation to disordered eating, and in relation to body shame, the present study furthers the research area by examining all variables within one study, i.e., examining the specific role of body shame within the relationship between online sexual harassment and disordered eating. In line with objectification theory (Fredrickson & Roberts, 1997), the findings from the present study and previous research suggest that women who are sexually harassed (whether that be in-person or online), may start to internalize these experiences and evaluate themselves based on cultural ideals. These women may then experience body shame due to the discrepancy between their real bodies and culturally idealized bodies, resulting in these women experiencing disordered eating attitudes and behaviors to try and reduce this discrepancy. However, due to the cross-sectional nature of the present study, longitudinal data are required to further test this indirect association and test true mediation within this online context. Furthermore, a direct relationship between online sexual harassment and disordered eating attitudes was also observed in the present study, suggesting that the relationship is not fully explained by body shame (Zhao et al., 2010). This may indicate that other factors relevant to objectification theory may play a role in this relationship, and may warrant further investigation, such as self-objectification, body surveillance, self-esteem, and personal safety anxiety (Dollimore et al., 2025; Hayes et al., 2021; Oliver et al., 2024).

### Limitations and Future Research

The present study has several limitations. First, we used a cross-sectional design to examine the direct and indirect relationships between online sexual harassment, disordered eating attitudes, and body shame in young women. Although the present study provides initial support for an indirect association, a longitudinal design is needed to fully test true mediation (Jose, 2016), to ensure temporal precedence, and to test whether changes in online sexual harassment experiences and body shame result in changes in disordered eating attitudes over time (Hayes et al., 2021; MacKinnon & Luecken, 2008).

Second, we relied on self-report measures. Acquiring accurate self-disclosures is challenging, due to the sensitive and potentially distressing nature of disclosing online sexual harassment experiences (Oliver et al., 2024), especially as research suggests that unwanted sexual experiences often go unreported (Brunton-Smith et al., 2022; Mulder et al., 2021). Moreover, the Online Sexual Harassment Scale only assessed online sexual harassment experiences in the past 12-month period. Previous research has suggested that historical in-person sexual harassment experiences predict disordered eating in undergraduate women (Harned, 2000). Therefore, future research should seek to examine historical experiences of online sexual harassment and how this relates to current disordered eating attitudes. Moreover, we relied on self-report screening for excluding participants with a diagnosed eating or body image disorder. Denial and distortion of experience can be characteristic of disordered eating presentations, as research suggests that 75% of eating disorder patients (and sub-clinical populations) deny they have a problem with eating (Howard et al., 2020; Schoen et al., 2012). This may pose a challenge for self-report screening; therefore, future research should consider alternative screening procedures (Vitousek et al., 1991).

Thirdly, we conducted our study online, via a survey platform. This method of data collection can be vulnerable to BOT (short for robot) responses (computer programs that automatically complete web-based surveys with random responses; Xu et al., 2022), which may threaten the integrity of the data (Irish & Saba, 2023; Storozuk et al., 2020). Therefore, future research should include the use of reCAPTCHA (a Turing test to discriminate humans from BOTs) to reduce BOT responses (Sherman et al., 2024).

Fourthly, the sample size (*N* = 146) is relatively small. Although the sample size exceeds estimates from the priori power analysis, this sample size may still limit the generalizability of the findings (Yang & Berdine, 2023). Future research should seek to recruit a larger sample, to reduce potential bias and increase the stability and generalizability of the findings (Schönbrodt & Perugini, 2013).

Finally, the sample in the present study was largely white and heterosexual, like previous work in the area (Iroegbu et al., 2024). However, research suggests that women from the LGBTQ+ community and ethnic minority communities may be more likely to experience online victimization (Iroegbu et al., 2024). Therefore, future research should seek to examine the relationships between online sexual harassment, disordered eating attitudes, and body shame in a more diverse sample of young women.

## Conclusion

The present study aimed to examine the direct and indirect relationships between online sexual harassment, disordered eating attitudes, and body shame in young women. We found that online sexual harassment positively predicted disordered eating attitudes and body shame positively predicted disordered eating attitudes. We also found an indirect association between online sexual harassment and disordered eating attitudes, via body shame. These findings offer initial support for examining the utility of online sexual harassment and body shame to further understand disordered eating in young women.
